# Single-Cell Mononucleotide Microsatellite Analysis Reveals Differential Insertion-Deletion Dynamics in Mouse T Cells

**DOI:** 10.3389/fgene.2022.913163

**Published:** 2022-07-08

**Authors:** Elli-Mari Aska, Bulat Zagidullin, Esa Pitkänen, Liisa Kauppi

**Affiliations:** ^1^ Research Program in Systems Oncology, Faculty of Medicine, University of Helsinki, Helsinki, Finland; ^2^ Institute for Molecular Medicine Finland (FIMM), HiLIFE, University of Helsinki, Helsinki, Finland; ^3^ Applied Tumor Genomics Research Program, Faculty of Medicine, University of Helsinki, Helsinki, Finland; ^4^ iCAN Digital Precision Cancer Medicine Flagship, Helsinki, Finland

**Keywords:** DNA mismatch repair, DNA replication, microsatellite, tissue-specific transcription, replication timing, repeat instability, deletions, insertions

## Abstract

Microsatellite sequences are particularly prone to slippage during DNA replication, forming insertion-deletion loops that, if left unrepaired, result in *de novo* mutations (expansions or contractions of the repeat array). Mismatch repair (MMR) is a critical DNA repair mechanism that corrects these insertion-deletion loops, thereby maintaining microsatellite stability. MMR deficiency gives rise to the molecular phenotype known as microsatellite instability (MSI). By sequencing MMR-proficient and -deficient (*Mlh1* ^*+/+*^ and *Mlh1* ^*−/−*^) single-cell exomes from mouse T cells, we reveal here several previously unrecognized features of *in vivo* MSI. Specifically, mutational dynamics of insertions and deletions were different on multiple levels. Factors that associated with propensity of mononucleotide microsatellites to insertions versus deletions were: microsatellite length, nucleotide composition of the mononucleotide tract, gene length and transcriptional status, as well replication timing. Here, we show on a single-cell level that deletions — the predominant MSI type in MMR-deficient cells — are preferentially associated with longer A/T tracts, long or transcribed genes and later-replicating genes.

## Introduction

Microsatellites, tandem repeat DNA sequences consisting of 1–6 nucleotide (nt) repeat units, are highly mutable, due to their propensity to form insertion-deletion (indel) loops during DNA replication. This can lead to insertions or deletions of repeat units, a phenomenon called microsatellite instability (MSI). Generally, instability increases as the length of the repeat increases, and nucleotide composition has also been shown to affect the stability of the microsatellite (Lujan et al., 2015).

Surveillance of DNA replication fidelity ensures normal and healthy propagation of cells. Genomic stability is maintained by multiple levels of repair, starting from DNA polymerases’ intrinsic proofreading activity and a post-replicative repair system called DNA mismatch repair (MMR). DNA replication initiates when replisomes are assembled at origins of replication, where each bi-directional replication fork starts to move along the DNA in opposite directions, and ends when adjacent replication forks fuse together (O’Donnell et al., 2013). DNA replication can be divided into distinct replication features (Rhind and Gilbert, 2013; Zhao et al., 2020). Initiation zones (IZ) are narrow regions containing the replication origin. Constant timing regions (CTRs) are large genomic segments that have the same replication timing window. CTRs can be divided into early and late CTRs, which are surrounded by timing transition regions (TTRs). TTRs are usually unidirectional and progress from early CTRs to late CTRs. Flanked by TTRs, replication regions called breakages likely indicate replication origin firing between the TTR slopes. Termination sites are locations where two replication forks fuse together (Rhind and Gilbert, 2013; Zhao et al., 2020). Occasionally, DNA polymerases make errors during the replication process. Multiple factors affect polymerase fidelity, from template sequence to transcriptional activity and the replication process itself.

The MMR system repairs base-base mismatches and small indel loops. An integral protein in the MMR process is MLH1, which is responsible for MMR initiation and recruitment of other repair proteins to the DNA lesion (Prolla et al., 1994). *Mlh1*-deficient cells cannot repair these errors and accumulate mutations in every cell division, leading to base substitutions and small deletions and insertions. DNA mismatch repair is recruited to chromatin by the histone 3 lysine 36 trimethylation (H3K36me3) mark enriched in exons of actively transcribed genes (Kolasinska-Zwierz et al., 2009; Li et al., 2013). This has been shown to decrease the local mutation rate on a mega-base scale, in exons versus introns, and in exons located in the 3′ ends of actively transcribed genes; (Supek and Lehner, 2015; Frigola et al., 2017; Huang et al., 2018; Aska et al., 2020). Replication timing has also been shown to affect MMR efficiency, with late replicating regions being more unstable than early replicating regions (Supek and Lehner, 2015).

DNA replication and transcription can take place contemporaneously in the same genomic location, leading to possible conflicts between these two machineries, which have been shown to be a significant source of genomic instability in cancer cells (García-Muse and Aguilera, 2016). Multiple possible mechanisms have been proposed to cause instability in replication-transcription conflicts. The replisome cannot move past the transcription machinery, leading to replication stalling and potentially to DNA damage. Head-on collisions have been shown to be more detrimental compared to co-directional collisions (Prado and Aguilera, 2005; Srivatsan et al., 2010). RNA-DNA hybrids formed during transcription have been shown to cause replication fork stalling in 3′ ends of genes transcribed by RNA polymerase II. All these scenarios can lead to replication conflicts and consequently to genomic instability (Skourti-Stathaki and Proudfoot, 2014). Temporal separation of replication and transcription in part mitigates conflicts between these two processes; genes expressed during early S phase are generally replicated in late S phase and vice versa (Meryet-Figuiere et al., 2014).

To elucidate *in vivo* microsatellite dynamics and genomic features that potentially affect MSI, we analyzed single-cell whole-exome data from *Mlh1* ^*−/−*^ and *Mlh1* ^*+/+*^ thymic T cells. By comparing MSI in normal (*Mlh1* ^*+/+*^) cells to that of MMR-deficient (*Mlh1* ^*−/−*^) cells, we can pinpoint where replication errors have occurred. We found A/T repeats of 10–14 nt length to be especially vulnerable to deletion accumulation, which decreases the number of such repeats compared to repeats in the reference genomic sequence. Insertions and deletions of repeat units affected either shorter, transcribed genes, or longer, silent genes. Genes enriched with deletions were generally replicated in a wider time window within S phase, while insertions were more common at microsatellites within genes that replicate earlier in S phase.

## Materials and Methods

### Single-Cell Whole-Exome Sequencing Data

VCF files containing indel variants in thymic T cells from *Mlh1* ^*+/+*^ (*n* = 22) and *Mlh1* ^*−/−*^ (*n* = 22) were produced and obtained from Aska et al. (2020). In brief, the data was produced from single thymic T cells collected from two 12 week old *Mlh1* ^*−/−*^ mice and two of their wildtype littermates. *Mlh1* ^*−/−*^ mice are DNA mismatch repair deficient and accumulate point mutations and small insertions and deletions in each cell replication. Single-cell genomes were isolated and amplified in the Fluidigm system, followed by whole-exome sequencing. Raw sequencing files were aligned to mouse genome version GRCm38/mm10 using bowtie2, followed by variant calling using GATK v3.8-0-ge9d806836 HaplotypeCaller. All variants observed close to the *Mlh1* gene (1.8 Mb window) were removed as those are likely to be artifacts arising from the *Mlh1* knock-out construct (Aska et al., 2020).

### Identification of Unstable Microsatellites

**FIGURE 1:**
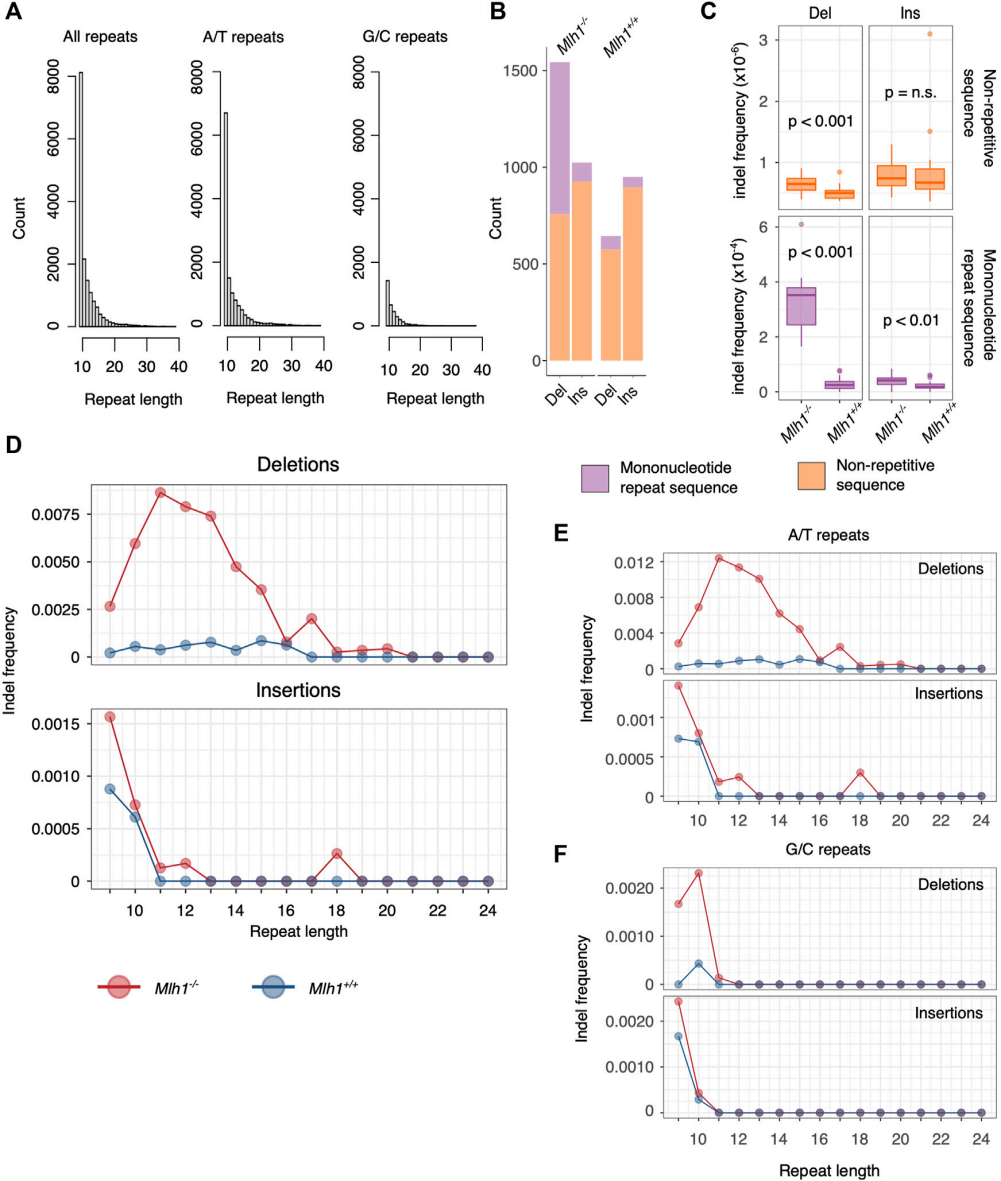
Deletions accumulate to 10–14 nucleotides long mononucleotide microsatellites. **(A)** Length distribution of mononucleotide microsatellites in the mouse exome. Shorter A/T repeats account for the majority of mononucleotide repeats. **(B)** Number of *de novo* mutations in mononucleotide repeats of length 9–24 nt versus non-repetitive sequences (all sequences that do not fulfill our criteria of a mononucleotide repeat). More *Mlh1* ^*−/−*^ deletions mapped to mononucleotide repeats than to non-repetitive sequences, whereas insertions were mainly found within non-repetitive sequences. In *Mlh1* ^*+/+*^ cells, the majority of indels accumulate in non-repetitive sequences. **(C)** Deletion and insertion frequencies in mononucleotide repeats were significantly higher in *Mlh1* ^*−/−*^ cells compared to *Mlh1* ^*+/+*^ cells, as was the deletion frequency within non-repeat associated sequences. Within non-repetitive sequences, no difference in insertion frequency was observed between *Mlh1* ^*−/−*^ and *Mlh1* ^*+/+*^ cells. *p* values from two-tailed Mann-Whitney U-test. **(D)** Indel frequencies at mononucleotide repeats of different lengths. 10–14 nt long mononucleotide microsatellites accumulated deletions in *Mlh1* ^*−/−*^ cells but not in *Mlh1* ^*+/+*^ cells. Insertions were mainly found in mononucleotide repeats of <11 nt length in both genotypes. **(E–F)** Indel frequency in A/T and G/C repeats. A/T repeats of length 10–14 nt long showed increased deletion burden in *Mlh1* ^*−/−*^ cells compared to *Mlh1* ^*+/+*^ cells. In E, note the different scales for deletion vs insertion frequencies. G/C repeats behaved visibly differently than A/T repeats, with increased deletion and insertion frequency in short repeats (<11 nt).

Microsatellites in the mouse exome were called as described in Aska et al., 2020. In brief, a FASTA file containing the sequence information of the mouse exome was analyzed for mononucleotide repeats using STR-FM in the Galaxy platform. For analyses presented here, the minimum length for mononucleotide repeat was set to be 9 bp and the maximum to be 24 bp. These thresholds for mononucleotide length were chosen based on mutability of different length mononucleotide repeats (Kondelin et al., 2017), as well as the overall abundance of different-sized repeats (Figure 1A). The following analyses were performed for all mononucleotide repeats, as well as A/T and G/C repeats separately, where applicable. Mononucleotide repeats that had insertions or deletions in *Mlh1* ^*−/−*^ or *Mlh1* ^*+/+*^ single-cell exomes were determined to be unstable repeats.

### Target Mononucleotide Repeat Length Analysis

We calculated the number of different length mononucleotide repeats in the mouse exome and analyzed the number of deletions and insertions stratified by mononucleotide repeat length and normalized it by the number of each mononucleotide repeat times the length of the repeat, respectively. For example, the number of deletions in 11 bp repeat was normalized by the number of 11 bp long repeats times 11 (the number of available nucleotides vulnerable for indels). The new repeat length was calculated by subtracting the size of the indel from the length of the repeat. The change in the numbers of different length repeats was calculated by dividing the number of different length repeats after insertion or deletion by the number of different length repeats in the reference repeat set (see Identification of unstable microsatellites). For example, after deletions, some of the 11 bp repeats have changed to 10 bp repeats. The new numbers for 11 bp and 10 bp repeats were counted and then divided by the counts of 11 bp and 10 bp repeats in the reference genome.

### MSI Target Gene Analysis

Each gene in the mouse exome was analyzed for MSI. First, indels were mapped to mononucleotide repeats, which were then denoted unstable microsatellites. These unstable microsatellite were then mapped to genes from the UCSC KnownGene track using an R package *VariantAnnotation.* The number of unstable microsatellites in a gene was normalized by the total number of microsatellites in the gene in question. Insertions and deletions in *Mlh1* ^*−/−*^ and *Mlh1* ^*+/+*^ single-cell samples were analyzed separately. All genes that showed MSI in at least one *Mlh1* ^*−/−*^ single-cell sample were considered to be target genes for DNA replication errors. We identified 661 genes with microsatellite deletions (MSI-del genes) and 88 genes with microsatellite insertions (MSI-ins genes). After excluding genes in chromosome X (due RepliSeq data used which only contains data from the autosomes, see Methods section *Replication timing features*), 624 MSI-del and 84 genes MSI-ins were further analyzed for replication timing. The transcriptional status of these genes was determined by the presence or absence of RNA Pol II at the affected gene. RNA Pol II ChIP-seq data (ENCFF918VSQ) was acquired from ENCODE in BED format.

### Mononucleotide Repeats in Genic Locations

The location of mononucleotide repeats of length 9 to 24 nt in different genic locations (coding, 5′UTR, 3′UTR, intron, promoter or intron-exon boundary) and their consequences to the reading frame of genes were analyzed using *VariantAnnotiation* R package with UCSC KnownGene track as a gene model. The same analysis was conducted for unstable mononucleotide repeats (see methods section *Identification of unstable microsatellites*). The number of unstable mononucleotide repeats in each genic location were pooled within each genotype and normalized by the total number of all mononucleotide repeats in each genic location, giving us an enrichment value for instability in a given location. Values <1 indicate fewer unstable repeats observed over analyzed single-cell exomes than the total number of repeats in a given location and values >1 indicate greater number of unstable repeats observed in a given genic location.

### Replication Timing Features

Replication timing data in mice was produced by Zhao et al. (2020) and downloaded from GEO repository (GSE137764). Replication timing data were preprocessed following Zhao et al. (2020). The data matrix was smoothed using a 2D Gaussian filter with s.d. = 1, then it was normalized column-wise such that 16 fractions in a single bin sum to 100, where each column (bin) refers to a 50 kb long genome region, and 16 fractions are the replication (S phase) timepoints. Six replication timing features were considered: initiation zones (IZs), large constant timing regions (CTRs), termination sites, timing transition regions (TTRs), breakage bins and other. The following modifications were introduced to the definitions of the timing features, as compared with Zhao et al.: termination sites can be up to 150 kb (3 bins) long; TTRs were pooled together with breakage bins as a single class. If the identity of a genomic bin was unclear it was labeled as “other”.

Genomic bins were classified into six replication timing feature classes. According to Brison et al. (2019) each genomic bin was assigned a numeric value (S_50_) denoting the point in S phase when 50% of cells finished replicating the corresponding sequence. It was calculated for each genomic bin independently using linear interpolation as implemented in *numpy* Python package, version 1.20.3. S_50_ ranges between 0 and 1, with low values indicating early S phase replication, and high values indicating late S phase replication. Genomic bins with missing values (2.35% across 19 chromosomes) were excluded from the analysis. The timing difference (S_diff_) between 75% and 25% of cells replicated, z-score normalized per chromosome with a cutoff of 1.65, was used to account for experimental noise.

### Statistical Testing

Statistical tests were performed using two-sided Mann-Whitney U-test or t-test for continuous values or χ^2^-test for counts and stated when applicable. Pearson’s product-moment correlation was used to quantify the linear relationship between replication timing, gene lengths and the fraction of mutated samples. Two-sided t-test was used to check for differences in replication timing, gene lengths and the fraction of mutated samples between MSI-ins and MSI-del genes stratified by their RNA Pol II status. Chromosome X genes were removed from the analysis. All statistical tests were performed in R.4.2.0. Relevant code and raw data can be found on: https://github.com/netphar/repliseq.

## Results

### Most Replication Errors Accumulate to A/T Mononucleotide Repeats

The mouse genome consists of 3–4% of microsatellites, and the most abundant microsatellite class is mononucleotide repeats (Komissarov et al., 2011). Most mononucleotide repeats are short, and the longer the repeat becomes, the less abundant it is in the mouse exome (Figure 1A, first panel). Overall, we analyzed 15266 mononucleotide repeats of 9–24 nt length that were found within the mouse exome capture regions, and further stratified them to repeats consisting of either adenine (A)/thymine (T) or guanine (G)/cytosine (C). A/T repeats make up the majority of mononucleotide repeats in the mouse exome (*n* = 12142), while G/C repeats are sparse (*n* = 3124) (Figure 1A, second and third panel).

To better understand the dynamics of contractions and expansions of these repeats, we mapped small insertions and deletions to mononucleotide microsatellites in the mouse exome using single-cell whole exome data of thymic T cells from *Mlh1* ^*−/−*^ and *Mlh1* ^*+/+*^ mice. By comparing the mutational profiles of *Mlh1* ^*−/−*^ and *Mlh1* ^*+/+*^ cells, we can directly delineate where replication errors occur since they are left unrepaired in *Mlh1* ^*−/−*^ cells. Moreover, analyzing where *de novo* mutations occur in *Mlh1* ^*+/+*^ cells can uncover what microsatellite features associate with sloppier MMR-mediated elimination of replication errors.

Patterns of deletions were different depending on MMR status of the cells. Firstly, the total number of deletions in *Mlh1-*deficient cells was higher than in wildtype cells (Figure 1B). Moreover, this difference arose particularly from deletions at mononucleotide microsatellites, which were substantially more frequent in *Mlh1* ^*−/−*^ cells (median = 0.000352 del/bp, IQR = 0.000136) compared to wildtype cells (median = 0.0000249 del/bp, IQR = 0.0000247) (*p* = 1.433 × 10^−8^) (Figure 1C). For microsatellite insertions, the difference in abundance between *Mlh1* ^*−/−*^ (median = 0.0000415 ins/bp, IQR = 0.0000228) and *Mlh1* ^*+/+*^ (median = 0.0000177 ins/bp, IQR = 0.0000140) cells was smaller yet significant (*p* = 0.00561) (Figure 1C). Deletions, but not insertions, at non-mononucleotide sequences showed a genotype dependent difference (*p* = 0.000194) (Figure 1C). In both genotypes, *Mlh1* ^*−/−*^ and *Mlh1* ^*+/+*^, the vast majority of insertions mapped to non-repetitive sequences (*Mlh1* ^*−/−*^: mean = 42.1, s.d. = 15.7, *Mlh1* ^*+/+*^: mean = 40.8, s.d. = 18.7), compared to mononucleotide repeats (*Mlh1* ^*−/−*^: mean = 4.62, s.d. = 2.27, *Mlh1* ^*+/+*^: mean = 2.52, s.d. = 1.29) (Figure 1B).

We then took a closer look at how mononucleotide repeat length affects microsatellite stability in the presence or absence of MMR. Interestingly, 10–14 nt long mononucleotide repeats were most prone to deletions in *Mlh1* ^*−/−*^ cells (Figure 1D), suggesting that such repeats represent a particular challenge to DNA polymerases. While deletions behaved genotype-dependently, insertions did not; in both genotypes, the most unstable class was mononucleotide repeats of <11 nt length (Figure 1D). A/T repeats (Figure 1E) followed the same pattern observed for mononucleotide repeats overall (Figure 1D) and likely drives this overall pattern due to their greater abundance. G/C repeats had a distinct instability signature: the most unstable repeats, in terms of both insertions and deletions, were 9–10 nt long (Figures 1E,F). Taken together, A/T repeats showed an increased deletion burden at longer mononucleotide repeats (11–14 nt) and insertional burden at shorter repeats (9–10 nt).

### MMR Deficiency Changes Microsatellite Length in a Stepwise Manner

**FIGURE 2:**
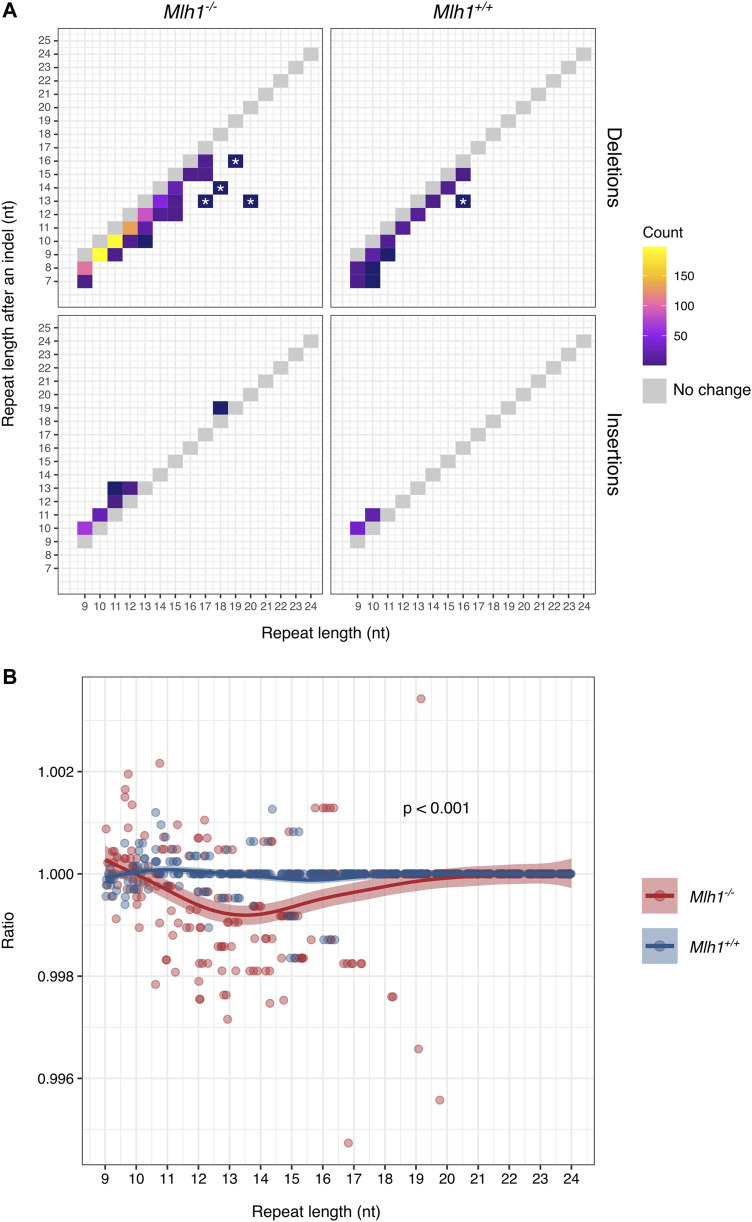
Mononucleotide microsatellites mostly lose single repeat units in a stepwise manner. **(A)** Changes in mononucleotide repeat length as a result of deletions (upper panel) and insertions (lower panel). Single-nucleotide shifts account for most indels, while >1-nt shifts are less common. Gray boxes indicate size of the unmutated repeat. Large shifts private for a single cell are marked with asterisks. **(B)** Change in mononucleotide repeat lengths after indels affects the exome-wide repeat length composition. In *Mlh1* ^*−/−*^ cells, the number of 10–14 nt long repeats decreased as compared to numbers observed in the reference genome, while 9-nt long repeats increased, compared to *Mlh1* ^*+/+*^ cells, where the mononucleotide repeat landscape remained relatively unchanged (*p* = 0.00030). Ratio depicts the number of observed repeats divided by the number of reference repeats for each different length mononucleotide repeats, value 1 indicating no change, and values <1 a decrease in the number of the repeats and values >1 an increase in the number of the repeats. The difference in the ratios was tested using a two-tailed Mann-Whitney U-test.

The difference in target repeat lengths in terms of contractions and expansions prompted us to further investigate how this phenomenon impacts the overall mononucleotide repeat composition of single-cell exomes. We analyzed how repeats of different lengths accumulate insertions and deletions, and the frequency of the resulting new lengths (after mutation). Most changes involved losses of single repeat units, and overall our data are consistent with single-unit stepwise microsatellite mutation (Figure 2A), in agreement with previous reports in MMR-deficient cells (Campregher et al., 2010; Shrestha et al., 2021). Interestingly, at longer (>15 nt) mononucleotide tracts, we also observed a few cases of deletions apparently involving multiple repeat units (marked with an asterisk in Figure 2A). These larger losses were observed only in A/T mononucleotide repeats and not in G/C repeats (Supplementary Figure S1A–B) and were private for a given single cell. Though rare, these mutations may hint at a distinct (non-stepwise) mutational process operating at longer repeat tracts.

Since not all repeats were equally unstable and deletions accumulated to repeats in a different fashion compared to insertions, we next took a look at how the mononucleotide repeat landscape changes due to contractions and expansions. Exome-wide, the number of 11–16 nt long repeats decreased and the number of 8–10 nt repeats increased in *Mlh1* ^*−/−*^ cells, as compared to *Mlh1* ^*+/+*^ cells where the balance remained unchanged (*p* = 0.000296) (Figure 2B). When A/T and G/C repeats were considered separately, most of the instability originated from A/T repeats rather than G/C repeats (*p* = 0.00055 and *p* = 0.30, respectively) (Supplementary Figure S2A–B). Here, we show evidence that MMR deficiency (accumulation of replication errors) increases the number of 9–10 nt repeats by insertions and decreases the number of longer repeats (11–19 nt) by deletions, modifying the single-cell microsatellite landscape.

### Distribution of Mononucleotide Indels Along Gene Bodies

**FIGURE 3:**
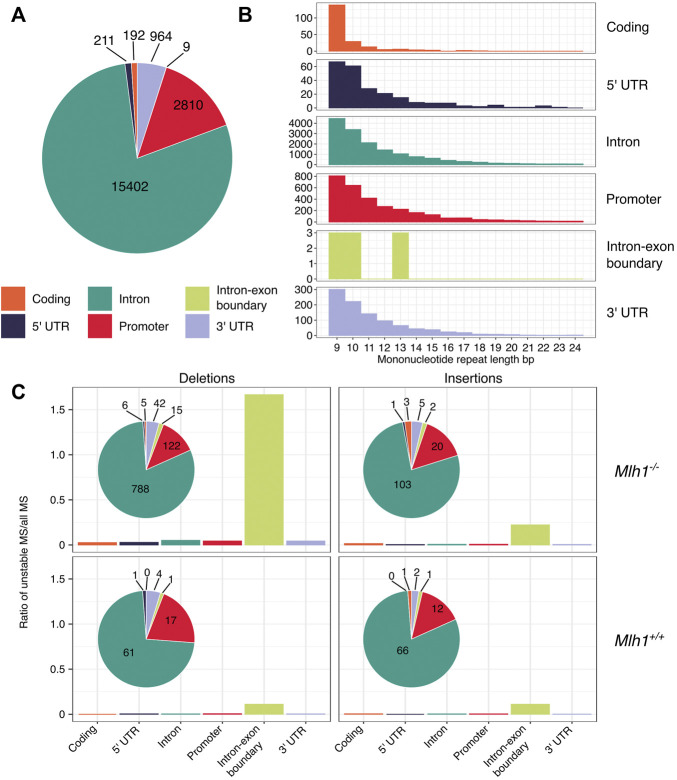
Deletions are enriched in mononucleotide repeats that are located within intron-exon boundaries. **(A)** Mononucleotide repeats in different genic regions. Exome-wide, the majority of mononucleotide repeats are located within intronic sequences. **(B)** Number of different-sized mononucleotide repeats in different genic regions. Coding sequences have relatively more short repeats when compared to other genic regions. **(C)** Indels in mononucleotide repeats within different genic regions. Deletions in mononucleotide repeats are enriched in exon-intron boundaries in *Mlh1* ^*−/−*^ cells, but not in *Mlh1* ^*+/+*^ cells. Insertions in mononucleotide repeats are not enriched in any particular genic region in *Mlh1* ^*−/−*^ or *Mlh1* ^*+/+*^ cells.

Whole-exome sequencing captures genomic sequences not only from coding regions, but also from parts of the surrounding non-coding regions (Guo et al., 2012), which allows us to examine how replication errors are distributed within genes (Aska et al., 2020, see Figure 1). The majority (79%) of the analyzed repeats resided within intronic sequences (Figure 3A). The next frequent location for mononucleotide repeats was promoter regions (14%), followed by 3′UTRs (5%), coding regions (1%), 5′UTRs (1%) and intron-exon boundaries (0.0005%) (Figure 3A). Upon closer examination of how different length repeats are distributed within genes, we discovered a difference between coding and non-coding sequences: mononucleotide repeats located within coding sequences tended to be shorter than repeats elsewhere (Figure 3B). Next, we analyzed how deletions and insertions in different-sized repeats were distributed along gene bodies. The total number of indels was highest within intronic repeats. Intron-exon boundaries appeared to be enriched with proportionately more deletions than other genic regions, particularly in *Mlh1* ^*−/−*^ cells, when normalized by the total number of mononucleotide repeats at intron-exon boundaries. However, the number of mononucleotide repeats that overlap with intron-exon boundaries in our dataset is very small (*n*= 9, Figure 3A) and this enrichment was not statistically significant. Nevertheless, the enrichment of deletions in these narrow regions of the exome raises the possibility that DNA polymerases struggle to replicate these regions (Figure 3C). These results indicate that microsatellites’ genic location also affects their stability.

### Deletions and Insertions Affect Different Genes

**FIGURE 4:**
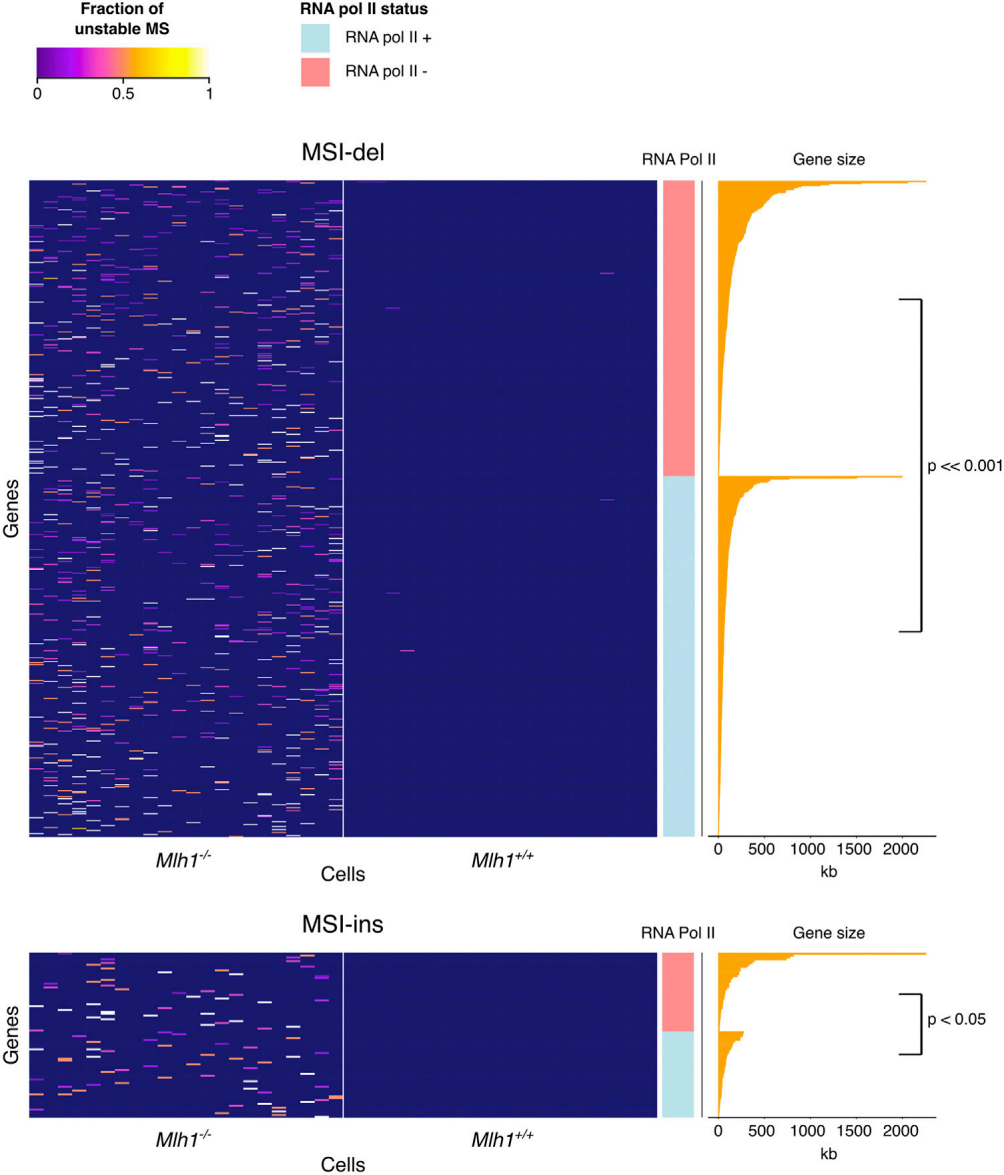
MSI in the mouse exome is enriched in long or transcribed genes. Heatmap of MSI frequency in the MSI target genes. MSI target genes are devoid of mutations in wildtype cells and enriched with either silent long genes or shorter, transcribed genes. Each column in the heatmap represents one single-cell sample of the genotypes indicated at the bottom. Rows are sorted by RNA Pol II status and gene length. Deletions (upper panel), but not insertions (lower panel), cluster to the same high-MSI genes across different *Mlh1* ^*−/−*^ cells.

Next, we analyzed all mouse genes for MSI and identified 661 genes that show MSI by deletions (MSI-del) and 88 genes by insertions (MSI-ins) in *Mlh1* ^*−/−*^ cells, but not in wildtype cells. All MSI genes identified here are listed in Supplementary Table S1. Previously, we showed evidence of differential instability of genes between *Mlh1* ^*−/−*^ and *Mlh1* ^*+/+*^ mice and found *Mcm7* and *Huwe1* to be targets for replication errors, both point mutations and indels (Aska et al., 2020). In addition to the previously observed overall high mutability of *Huwe1,* it emerged as one of the MSI-del genes. *Huwe1* is positive for H3K36me3 (Aska et al., 2020), a histone mark that facilitates the recruitment of the MMR system to the chromatin (Li et al., 2013). *Huwe1* is important for maintaining normal development of the T cell lineage (King et al., 2016), conceivably making this gene worthy of more stringent surveillance against replication errors in thymocytes. With only a few exceptions, the genes targeted for deletions (MSI-del) or insertions (MSI-ins) in *Mlh1* ^*−/−*^ cells were devoid of indels in *Mlh1* ^*+/+*^ cells (Figure 4). These MSI-del and MSI-ins genes were also longer (MSI-del genes: mean = 126 kb, *p* < 2.2 × 10–^16^; MSI-ins genes: mean = 142 kb, *p* = 0.00024) than randomly selected genes on average (Figure 4). In *Mlh1* ^*−/−*^ cells, MSI target genes were enriched for transcriptionally active genes: 55% of MSI-del genes, *p* < 2.2 × 10^−16^, and 52% of MSI-ins genes, *p* = 0.0032 were marked with RNA Pol II (Figure 4). Of all genes in mouse thymus, 36% are RNA Pol II positive. The effect of MSI on gene function depends on the location of the event in the gene: deletions or insertions that are located within coding regions can lead to frameshift mutations, while such events occurring within other regions of genes are likely to be less detrimental. Only six out of 661 (∼1%) MSI-del genes and three out of 88 (∼3%) MSI-ins genes carried frameshift mutations. This is not surprising given the fact that only 1% of the analyzed mononucleotide repeats fall within the coding sequence. Even though the number of genes with frameshift-causing mutations was low in our data set, the fact that certain genes constitute “MSI hotspots” makes them prone to accumulate potentially deleterious replication errors over time. These results reveal how replication errors, specifically deletions, tend to accumulate in certain genes, and these are (in wildtype cells) efficiently repaired by MMR.

### Differential Accumulation of Insertions and Deletions Correlates With Replication Timing

**FIGURE 5:**
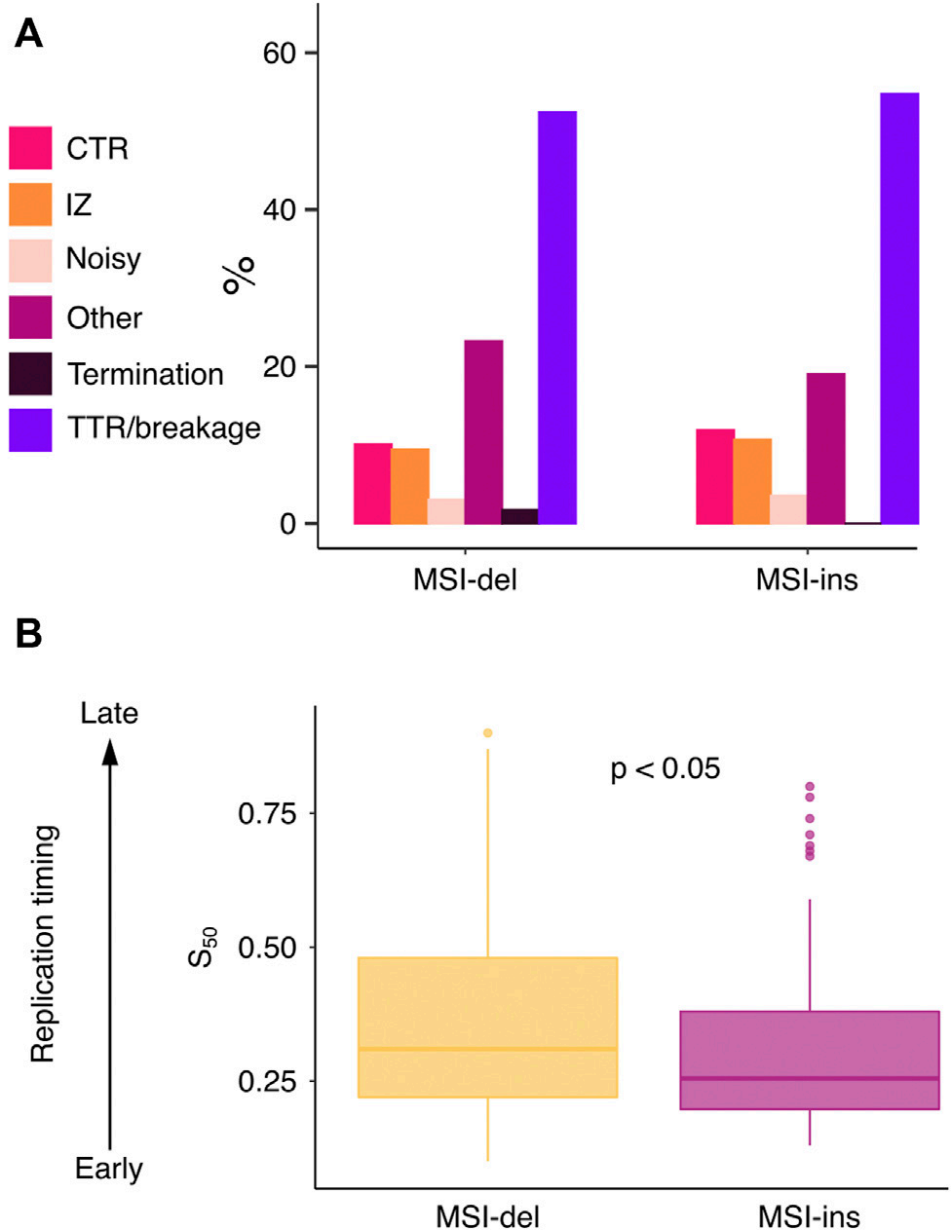
Deletions and insertions are associated with differential DNA replication time windows. **(A)** Barplot of the number of replication features identified at MSI target genes. **(B)** MSI-del genes are replicated later in S phase compared to MSI-ins genes (*p* = 0.023). S_50_ values were tested using two-tailed Mann-Whitney U-test.

Next, we analyzed how different replication features and replication timing associate with MSI-del and MSI-ins enriched genes. Replication timing has been shown to affect mutation rate and MMR efficiency in different cancers (Stamatoyannopoulos et al., 2009; Woo and Li, 2012; Supek and Lehner, 2015), late-replicating regions being more vulnerable to accumulating mutations. However, replication does not progress in a similar fashion around the genome. Rather, depending on the genomic segment, different replication features can be found (Zhao et al., 2020). We analyzed the frequency of replication initiation zones (IZ), timing transition regions (TTR), breakages in TTRs, constant timing regions (CTR) and replication termination sites (TS) (Supplementary Figure S3A) genome- and exome-wide using a published replication timing data set from mouse neural progenitor cells (Zhao et al., 2020). The frequency of different replication features was similar genome versus and exome-wide (Supplementary Figure S3B). The majority of MSI target genes (for both indel types) accumulated to TTRs/breakages and to genomic segments classified as “other”, meaning they could not be assigned to any particular feature (Figure 5A). Next, we examined general replication timing of MSI target genes and found a difference in timing between genes that accumulate deletions versus genes that accumulate insertions. The MSI-ins genes were mostly replicated earlier in S-phase (median S_50_ = 0.255, IQR = 0.182), while MSI-del genes were preferentially replicated later (median S_50_ = 0.31, IQR = 0.26) (*p* = 0.023) (Figure 5B).

We examined 84 MSI insertion and 624 deletion genes for associations between four genomic features, namely, replication timing, gene size, fraction of mutated samples and RNA Pol II status. Using Pearson’s r to measure the correlations among the first three genomic features we found that replication timing positively correlated with gene length in both MSI-ins and MSI-del genes (r = 0.51 with *p* = 6.01 × 10^−7^ and r = 0.29 with *p* = 2.65 × 10^−13^, respectively), meaning that longer MSI-ins and MSI-del genes tend to replicate later. Further, we compared replication timing, gene size and the fraction of mutated samples in MSI-ins and MSI-del genes stratified by RNA Pol II status. RNA Pol II positive MSI-del genes were shorter than RNA Pol II negative ones (*p* = 8.97 × 10^−6^), with median lengths of 52 Kb and 79 kb, respectively (Figure 4). RNA Pol II positive MSI-ins genes were shorter than RNA Pol II negative ones with median lengths of 57 Kb and 72 kb, respectively (*p* = 2.271 × 10^−2^) (Figure 4). RNA Pol II positive genes were characterized by earlier replication timing compared to RNA Pol II negative genes in the MSI-del subgroup, with S_50_ values of 0.32 vs. 0.41 (*p* = 6.70 × 10^−10^), whereas in the MSI-ins subgroup S_50_ values were 0.29 and 0.36 with *p* = 0.047). S_50_ denotes a normalized fraction of the S phase when 50% of cells have finished replicating. Lastly, we found that the frequency of mutated single cells in each gene did not correlate with replication timing or with RNA Pol II status in either MSI-del or MSI-ins subgroups.

## Discussion

By analyzing single-cell whole exome data from MMR-deficient and -proficient murine T cells, we show how mononucleotide stability is shaped by both replication errors and their repair by DNA mismatch repair. We provide evidence of how nucleotide composition, genic location, transcription, and replication timing affect the MSI landscape in mouse thymic T cells.

Our key finding was that *de novo* microsatellite insertions and deletions display distinct behavior that manifested itself in several ways. To some extent, this is likely due to their different origin: insertions arise from the nascent strand looping out during DNA replication, while deletions arise from the template looping out. In *Mlh1* ^*−/−*^ cells — where replication errors are left unrepaired — mononucleotide deletions were substantially more common than insertions (Figures 1B,C), which is in line with previous findings in MMR-deficient tumor genomes (Kondelin et al., 2017) and implies that microsatellite sequences are much more prone to template-strand loops compared to nascent-strand loops. Since mononucleotide deletions are so infrequent in wildtype cells (Figures 1B,C), we extrapolate that as many as >90% of template-strand loops may be recognized and corrected by MMR, preventing them from becoming permanent mutations. DNA mismatch repair has been shown to process 1 nt flaps generated during Okazaki fragment maturation (Kadyrova et al., 2015). In addition, studies conducted in *Schizosaccharymyces pombe* and *Saccharomyces cerevisiae* indicate that single-stranded replication gaps and Okazaki fragment abundance increase as replication proceeds (Daigaku et al., 2017; Koussa and Smith, 2021). We speculate that it may be the lagging strand that is more prone to template strand loops, in particular in the single-stranded regions between Okazaki fragments. This scenario could help explain why deletions tend to occur in later-replicating regions compared to insertions (Figure 5B)—presumably later in S phase the levels of single-stranded regions are elevated also in mouse.

Analysis of tumor whole-genome sequencing data by the Pan-Cancer Analysis of Whole Genomes (PCAWG) Consortium identified two indel mutational signatures associated with replication slippage, ID1 (1-bp insertions at 5+ bp long T mononucleotide tracts) and ID2 (1-bp deletions at 6+ bp long T mononucleotide tracts) (Alexandrov et al., 2020). *De novo* mutations in our exome sequencing data, obtained from non-malignant MMR-deficient mouse cells, are consistent with these two mutational signatures. By comprehensive characterization of insertion and deletion distributions across different-sized mononucleotide repeats (Figures 1D–F), we are now able to more precisely delineate the MSI signature of MMR-deficient cells: it consists of 1-bp deletions at 10–14 nt long A/T mononucleotide microsatellites. Insertions, consisting mostly of single-base expansions at 9-nt long repeats, were seen also in MMR-proficient cells, and therefore this signature cannot be attributed to defective post-replicative repair alone.

Deletions clustered into “universal” hotspots at the gene level, i.e., deletions hit the same gene in multiple single cells and in both *Mlh1* ^*−/−*^ animals analyzed (Figure 4). This kind of evidence for gene-level recurrent MSI was scarce for insertions, which appeared to be mostly stochastically distributed, typically hitting a given gene only once and in one individual cell (Figure 4). Probably this is largely due to the fact that our data set, in which only 9+ nt mononucleotide tracts were considered, contained so many fewer insertions—nearly an order of magnitude less than deletions. Based on the data analyzed here, it is not possible to say whether recurrent gene-level MSI hotspots exist for insertions. Dissecting this aspect of insertion dynamics would require sequencing of more cells and/or inclusion of shorter mononucleotide tracts in the analysis, so that more microsatellite insertion events would be captured.

Previous studies have shown genes with active transcription to be vulnerable for replication errors and targeted for H3K36me3-guided MMR protection (Li et al., 2013; Huang et al., 2018; Aska et al., 2020), consistent with the findings presented here. We found genes with increased MSI to be longer than genes on average in the mouse exome (Figures 4A,B). This cannot be explained by the higher abundance of microsatellites (i.e., more potential MSI targets) in longer genes, since MSI was corrected for the number of total mononucleotide loci in each gene. Instability appears to be driven either by active transcription or large gene size: RNA Pol II positive genes are shorter, while the longest MSI target genes were RNA Pol II negative. In terms of replication timing, we observed a difference between MSI-del and MSI-ins genes. Replication of MSI-del genes spans a wider time window in S phase and occurs on average with a delay compared to MSI-ins genes (Figure 5B), suggesting again a difference in the etiology of insertions and deletions. This to our knowledge is the first evidence of differential dynamics of insertions and deletions as a function of replication timing.

This study elucidates the multi-faceted differences in mutational dynamics of microsatellite insertions versus deletions in the mouse exome. We show, at single-cell resolution, that the propensity of mononucleotide microsatellites to insertions versus deletions is linked to microsatellite length, nucleotide composition of the mononucleotide tract, transcriptional status, as well as replication timing. Namely, deletions—the predominant MSI type in MMR-deficient cells—preferentially associate with longer repeats, A/T tracts and later-replicating genes. MSI-prone genes were typically longer than average and/or enriched with transcriptional activity. Jointly, the aforementioned features and their interplay contribute to region- and gene-specific vulnerability to MSI, a hallmark molecular phenotype of MMR-deficient cells and cancers. Future studies that focus on these factors specifically in the context of exonic (i.e., frameshift-causing) MSI may shed light on why MMR deficiency is tumorigenic only in certain tissues.

## Data Availability

Publicly available datasets were analyzed in this study. Single-cell whole exome sequencing data is available in FASTQ format and can be found in Sequencing Read Archive (SRA: PRJNA575619) (https://www.ncbi.nlm.nih.gov/sra). Publicly available RepliSeq data was acquired from GEO depository (GSE137764) (https://www.ncbi.nlm.nih.gov/geo/) and RNA pol II ChIPSeq (ENCODE: ENCFF119XEH) from ENCODE database (https://www.encodeproject.org/).
