# Supplementary material for: Single-Cell Mononucleotide Microsatellite Analysis Reveals Differential Insertion-Deletion Dynamics in Mouse T Cells
Source: Front Genet. 2022 Jul 8;13:913163. doi: 10.3389/fgene.2022.913163 (PMC9304711; doi:10.3389/fgene.2022.913163)
Supplement: Supplementary file 1 [file DataSheet1.pdf]

SUPPLEMENTARY

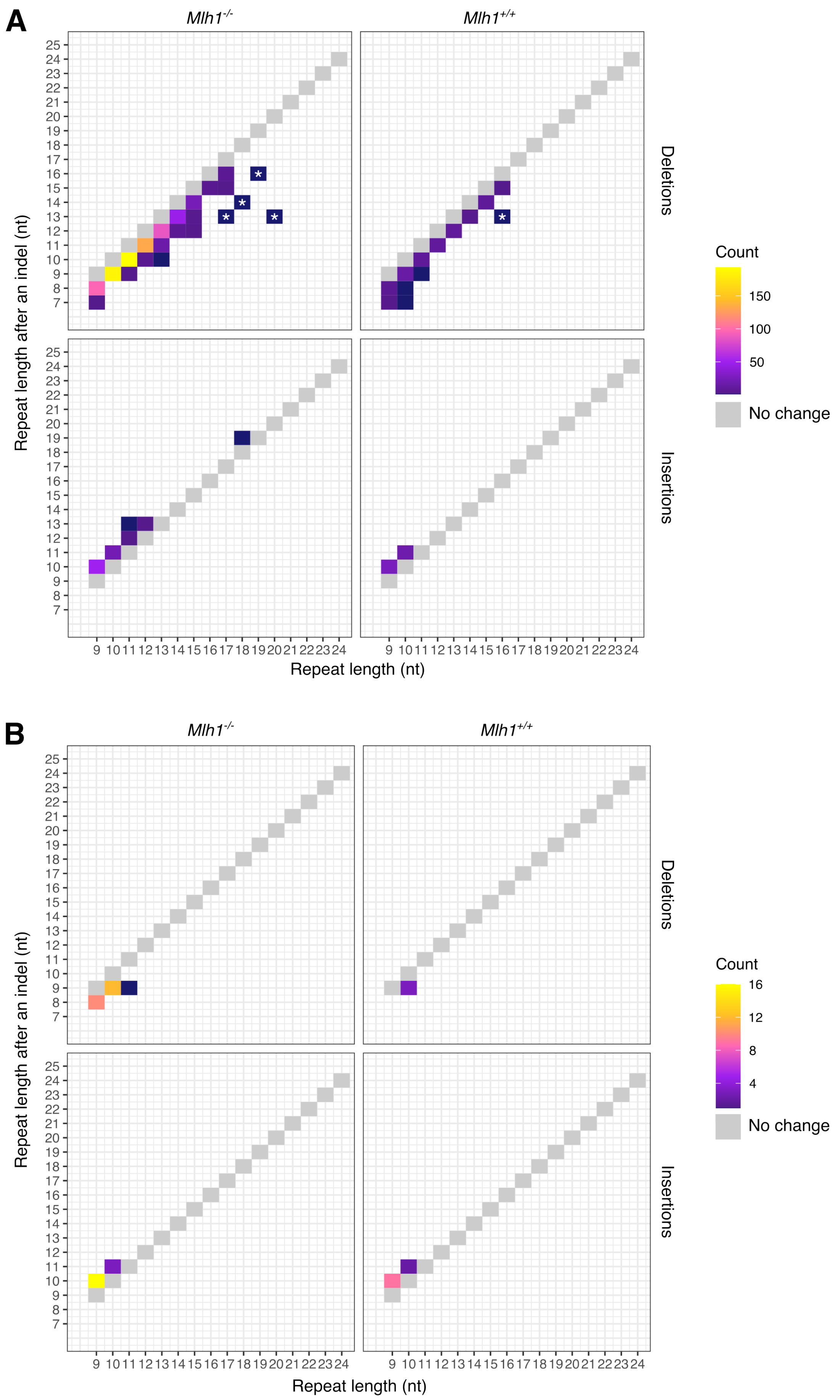
A
B
Mlh1-/-
Mlh1+/+
Deletions
Insertions
Count
150
100
50
16
12
8
4
No change
Repeat length after an indel (nt)
Repeat length (nt)
*

Supplementary Figure S1. Changes in A/T and G/C mononucleotide repeat lengths. Heatmap shows the frequency of lost or gained repeat units at A/T repeats (A) and G/C repeats (B). Large sifts private for a single cell are marked with a white asterisks.

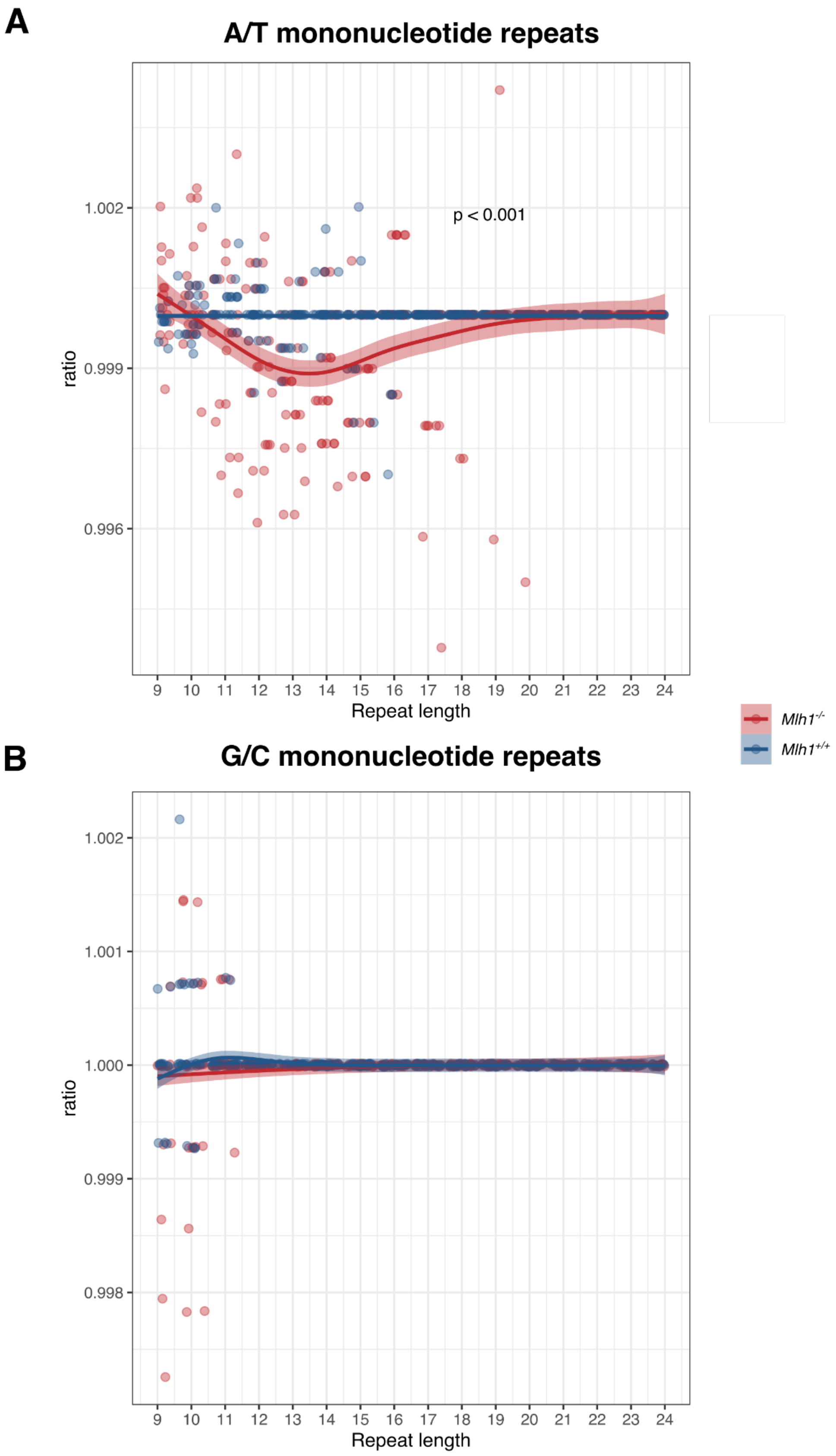

Supplementary Figure S2. Ratio of new repeat lengths and repeat lengths in mouse reference genome at A/T and G/C mononucleotide repeats. A) Indels at A/T repeats in *Mlh1*$^{-/-}$ cells change the repeat length composition in mouse exome when compared to ratios in *Mlh1*$^{+/+}$ cells (p =

0.00055) while B) the length composition of G/C repeats does not differ between *Mlh1*$^{-/-}$ and *Mlh1*$^{+/+}$ samples (p = 0.30). Each dot represents the ratio of observed and reference genome length mononucleotide repeats in each sequenced single-cell exome of the indicated genotype. The difference in ratios was tested using a two-tailed Mann-Whitney U-test.

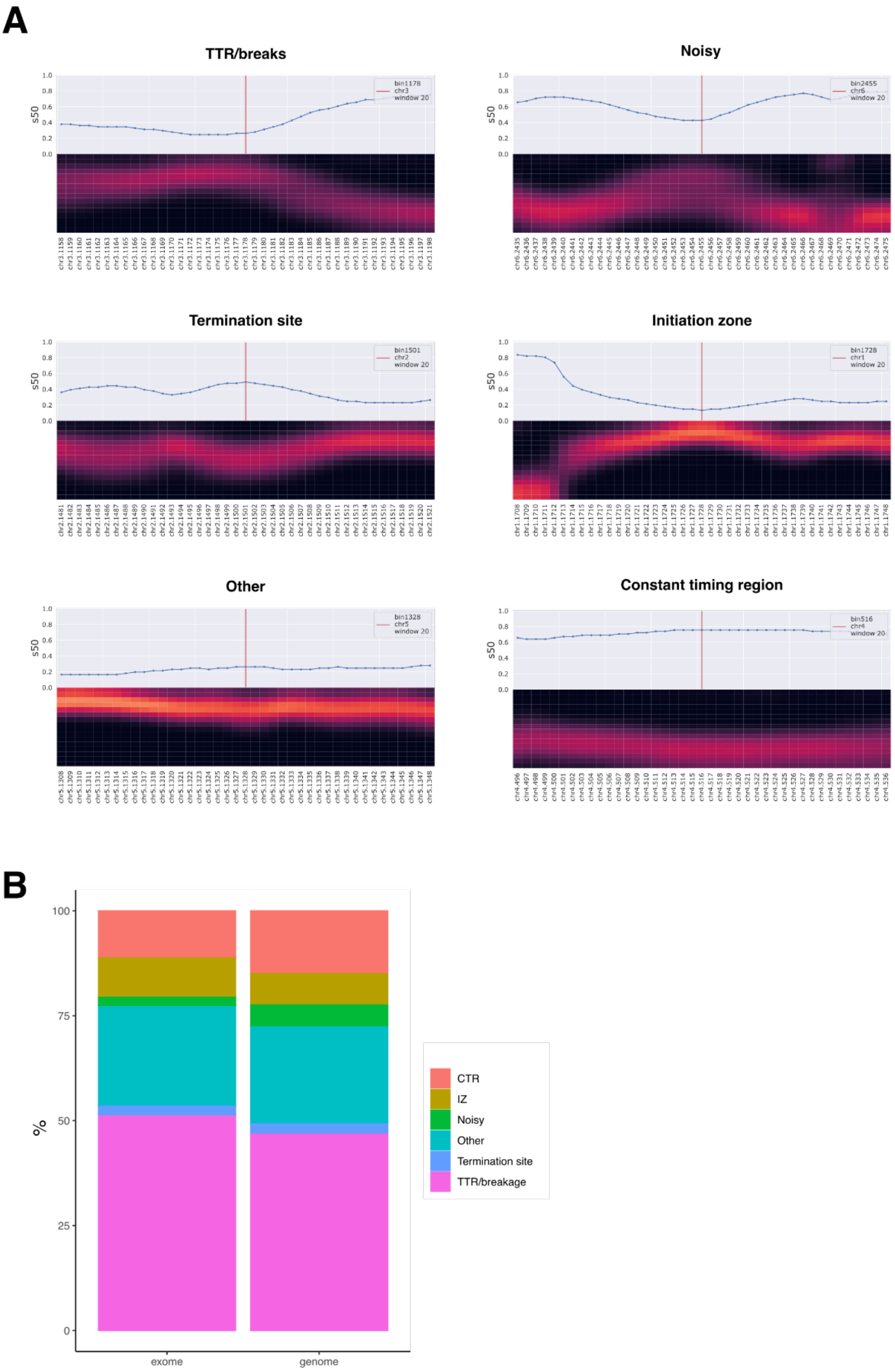
A
TTR/breaks
bin1178
chr3
window 20
Noisy
bin2455
chr6
window 20
Termination site
bin1501
chr2
window 20
Initiation zone
bin1728
chr1
window 20
Other
bin1328
chr5
window 20
Constant timing region
bin516
chr4
window 20
s50
1.0
0.8
0.6
0.4
0.2
0.0
B
%
100
75
50
25
0
exome
genome
CTR
IZ
Noisy
Other
Termination site
TTR/breakage

Supplementary Figure S3. Replication features in the mouse genome. A) Example figures of different replication features. B) Frequency of different replication features genome- and exome-wide.
